# Effects of breast size on breast reconstruction in BRCA mutation carriers and genetic high-risk patients after bilateral mastectomy

**DOI:** 10.1007/s12282-025-01691-w

**Published:** 2025-03-20

**Authors:** Martin C. Lam, Vendela Grufman, Sonia Fertsch, Florian Recker, Nicole E. Speck, Jian Farhadi

**Affiliations:** 1 Plastic Surgery Group, Zurich, Switzerland; 2 Division of Plastic, Reconstructive and Aesthetic Surgery, Department of Surgery, University Hospital Bonn, University of Bonn, Bonn, Germany; 3 Department of Plastic, Reconstructive and Aesthetic Surgery, Sana Hospital Düsseldorf, Düsseldorf, Germany; 4 Faculty of Health, University of Witten-Herdecke, Witten, Germany; 5 Department of Gynecology and Obstetrics, University Hospital Bonn, University of Bonn, Bonn, Germany; 6 Department of Plastic, Reconstructive, Aesthetic and Hand Surgery, University Hospital Basel, Basel, Switzerland; 7 Faculty of Medicine, University of Basel, Basel, Switzerland

**Keywords:** Bilateral breast reconstruction, Risk-reducing mastectomy, Autologous breast reconstruction, Implant-based breast reconstruction, Effects of breast size, BRCA mutation carriers, Hereditary breast cancer, Patients with high-risk breast cancer

## Abstract

**Background:**

Women with genetic susceptibility to breast cancer and indication for bilateral mastectomy are more likely to undergo implant-based breast reconstruction (IBR) than autologous breast reconstruction (ABR), while the impact of breast size in this context is insufficiently studied. Ultimately, comparative data on IBR and different types of ABR beyond abdominal-based flaps in genetic susceptible women remain scarce. This study aimed to evaluate factors associated with ABR and the effects of breast size for bilateral reconstruction in high-risk patients.

**Methods:**

A 2.5-year retrospective study was conducted at a single institution including all genetic high-risk patients who underwent bilateral mastectomy and breast reconstruction. Patients were stratified into two groups based on the weight of the mastectomy specimen. Small breast sizes were defined by mastectomy weights below 400 g, and medium-to-large breasts by specimen weights above 400 g. Binary logistic regression was performed to assess variables predictive of ABR, followed by an analysis of the breast size-dependent reconstructive algorithm and its complication rates.

**Results:**

We included 71 patients with BRCA1/2 (97.2%), CHEK2 (1.4%), and PALB2 (1.4%) mutations in the study. Among those, 68 IBRs and 74 ABRs were performed. Increasing age, immediate reconstruction, and medium-to-large breast size were predictive of ABR compared to IBR (*p* < 0.05).

In the IBR-group, the majority of preoperative small breasts received subpectoral implant placements (81.0%, *p* = 0.003), while prepectoral implants (53.9%, *p* = 0.003) were preferred in medium-to-large breasts. In the ABR-group, the deep inferior epigastric artery (DIEP) flap was the choice in the vast majority of cases with larger breasts (86.4%, *p* < 0.001), whereas the transverse myocutaneous gracilis (TMG) flap (46.7%, *p* < 0.001) and superior gluteal artery perforator (SGAP) flap (20.0%, *p* = 0.002) were only considered in small-breasted patients. No elevated incidence of overall complications with increasing breast size was found. However, patients with larger breasts were more likely to undergo elective revisions after IBR (*p* < 0.001) as well as ABR (*p* = 0.013). With regard to two-stage tissue expander reconstructions, high-risk patients with larger breast size revealed increased explantations (*p* = 0.043) and expander-related revisions requiring additional surgery (*p* = 0.003). The latter was significantly reduced by reduction mammoplasty prior to expander placement (*p* = 0.036).

**Conclusions:**

The preoperative breast size of gene mutation carriers influences the postmastectomy reconstructive choice. TMG and SGAP flaps are suitable options for bilateral reconstruction of genetic susceptible patients with small breasts, while DIEP flaps are preferred in larger breast sizes. With increasing breast size an elevated risk for elective revisions after either IBR or ABR need to be considered. Women with medium-to-large breasts exhibit increased morbidity related to expansion and genetic high-risk patients may benefit from prior reduction mammoplasty.

## Introduction

Breast cancer (BC) remains the most frequent malignancy in women worldwide [1, 2]. Genes most commonly mutated in hereditary BC are BRCA1/2, PTEN, and TP53, among many others, which make up to a lifetime risk of 80% for developing BC [1]. Other rare mutations, e.g., CHEK2, ATM, and PALB2, each have been associated with a doubled risk for BC [1]. BC of genetically susceptible patients often presents more aggressive growth, poor tumor differentiation, and worse prognosis than in non-mutation carriers [2]. High-risk patients without diagnosis of BC have to make the difficult decision between following strict screening guidelines, or undergoing prophylactic mastectomy [2]. The majority of genetically susceptible women choose to undergo prophylactic mastectomy, and the diagnosis of BC has shown to influence whether or not a subsequent breast reconstruction is considered [3, 4]. While a considerable amount of BRCA1 and BRCA2 mutation (BRCA1/2^Mut+^) carriers still undergo risk-reducing mastectomy (RRM) only, the number of patients with genetic susceptibility to BC who elect reconstruction after bilateral prophylactic mastectomy (BPM) has increased over the last decade [2–4].

The vast majority of genetically susceptible patients undergo implant-based breast reconstruction (IBR), whereas the amount of autologous breast reconstructions (ABR) remains inferior [2, 4]. Recent data on trends in breast reconstruction after BPM reveal that patient demographics correlate with the reconstructive outcome and technique, while the preoperative breast size remained unconsidered [2]. The breast size is associated with the body mass index (BMI), and both factors play relevant roles in the decision-making process for the type of reconstruction, as they affect the body constitution and donor-site suitability for microvascular breast reconstruction [5–7]. The preoperative breast size, stratified by mastectomy weight, has been shown to be a relevant parameter for selecting the suitable reconstruction. Patients with mastectomy weight below 400–500 g have been considered as small-breasted, above this range up to 1000 g as medium or intermediate, and above 800–1000 g as large [8–10]. Previous data suggested that small breast size was associated with IBR, patients with medium breasts were more likely to undergo transverse rectus abdominis myocutaneous (TRAM)/deep inferior epigastric artery perforator (DIEP) flap reconstruction, and large-breasted patients were most likely to undergo latissimus dorsi reconstruction [8]. However, reconstructive flap options and microsurgical procedures have evolved over the last decade, leading to a broadening of the range beyond abdominal-based and pedicled flaps toward a more individualized postmastectomy reconstruction according to the donor-site tissue availability [7, 11–13]. Obesity is seen as a pertinent risk factor demonstrating elevated complications in both IBR and ABR [14]. While previous studies favor pedicled flaps in obese patients, new data do not reveal increased failure rates in microvascular breast reconstruction and support free flap reconstructions independently of the BMI [15].

Ultimately, BPM is considered as elective surgery and subsequent breast reconstruction in these circumstances is associated with high patient expectations on the safety of the procedure and aesthetic outcome [16]. However, comparative data on different types of breast reconstruction investigating the applicability of non-abdominal flap in high-risk patients remain limited. As preoperative breast size has shown to influence the reconstructive algorithm in general, this study aimed to investigate its impact on the selection of postmastectomy bilateral breast reconstruction in this specific patient group, followed by an analysis of the complication rates. The secondary aim of the study is to provide valuable data on different breast reconstructive options for improved shared decision-making of high-risk patients and treating physicians.

## Patients and methods

### Patients

A retrospective review was conducted of all cases of IBR and ABR performed at a single institution (Plastic Surgery Group, Prof. Dr. Jian Farhadi, Zurich, Switzerland) between April 2018 and September 2020. The study specifically focused on BRCA1/2^Mut+^ carriers and other genetic susceptible patients that underwent bilateral mastectomy and reconstruction.

For patients with large and ptotic breasts, elective reduction mammoplasty (RM) prior to prophylactic mastectomy was offered and performed exclusively on cancer-free breasts, contingent upon the patient’s informed consent. All reconstructive options were discussed, and comprehensive preoperative informed consent for the surgical procedures was obtained. The selections of flaps were determined based on the donor-site suitability [7], with the DIEP flap being the preferred option in cases where an abdominal donor-site was available [12]. For patients lacking a suitable abdominal donor-site, alternative options, such as the upper thigh or gluteal region, were chosen based on patient preference.

Patients were categorized into two primary groups: the IBR-group and the ABR-group. An initial comparison was performed based on cancer status. Further stratification was undertaken by categorizing patients according to their mastectomy specimen weight, with small breasts defined by specimen weights below 400 g, and medium-to-large breasts by specimen weights exceeding 400 g.

### Assessments

Patient-specific data were analyzed, including routine preoperative breast measurements obtained prior to surgery. Cancer status and history of BC treatment were assessed, encompassing prior breast surgeries, type of mastectomy performed, and the administration of neoadjuvant or adjuvant therapies. Additionally, cases involving prior tissue expander (TE), direct-to-implant (DTI), immediate flap, and nipple–areola complex (NAC) reconstruction were identified.

Reconstruction procedures were categorized as follows: DTI and immediate flaps were classified as immediate reconstructions. Patients who underwent TE-based reconstruction were grouped as delayed–immediate, while cases without immediate breast reconstruction or those involving failed IBR were categorized as delayed. For comparative purposes, delayed–immediate and delayed reconstructions were consolidated into a single “delayed(-immediate)” group. Further details, including implant volume, type of implant pocket used, application of acellular dermal matrix (ADM), and type of free flap utilized, were thoroughly evaluated. During follow-up, Baker grade 3 or 4 capsules were counted as capsular contracture. Surgical outcomes were analyzed, incorporating all postoperative complications and elective revisions. The mean follow-up duration for the study cohort was 21.7 ± 7.0 months.

### Statistical analysis

Data collection was performed using an Excel spreadsheet (Microsoft Corporation, Redmond, WA, USA), and statistical analyses were conducted with SPSS Statistics, Version 25 (IBM Corp, Armonk, N.Y., USA). Categorical variables were analyzed using the Chi-square test, while continuous variables were compared using the *t* test, where applicable.

Binary logistic regression was conducted with ABR as dependent variable. Independent variables included age (in years), immediate reconstruction (reference category [RC]: no), medium-to-large breast size (RC: no), postmastectomy radiotherapy (PMRT; RC: no), nipple-sparing mastectomy (NSM; RC: no), and BC diagnosis (RC: no). The statistical significance level was set to *p* < 0.05. Results are reported as means with range or means ± standard deviation (SD).

## Results

### Patients

Over a 2.5-year period, 204 patients underwent IBR and 238 received free flaps. Among these, 71 carriers of genetic mutations carriers were identified and included in the study. The final study cohort comprised a total of 142 breast reconstructions. Of these, 69 patients were BRCA1/2^Mut+^ carriers (97.2%). All patients in the IBR-group (34 patients; 68 reconstructions) were BRCA1/2^Mut+^ carriers. In the ABR-group (37 patients; 74 reconstructions), two additional patients were included: one with a CHEK2 mutation and another with a PALB2 mutation.

The majority (*n* = 49, 69.0%) had a current or past diagnosis of BC, while less than one-third (*n* = 22, 31.0%) were genetically susceptible individuals without history of BC. Within the BC group, patients with IBR were significantly younger than those undergoing flap reconstruction. Although not statistically significant, the mean age of cancer-free patients with IBR was lower than that of the ARB-group (Table 1**)**.

**Table 1:** Patient-specific data of BRCA1/2^Mut+^ carriers and other genetic susceptible patients with and without diagnosis of breast cancer

	Patients with diagnosis of breast cancer (*n* = 49)			Patients with no breast cancer (*n* = 22)		
	IBR- group (*n* = 22)	ABR- group (*n* = 27)	*p* value	IBR- group (*n* = 12)	ABR- group (*n* = 10)	*p* value
Age (years)	41.9 (28–56)	50.6 (34–75)	**0.002**	40.3 (21–65)	47.7 (28–58)	0.152
BMI, *kg/m*^*2*^	22.6 (18–31)	24.7 (19–32)	0.055	22.7 (17–40)	25.9 (18–35)	0.217
BRCA 1, *n* (%)	11 (50.0)	18 (66.7)	0.238	4 (33.3)	5 (50.0)	0.429
BRCA 2, *n* (%)	11 (50.0)	8 (29.6)	0.145	8 (66.7)	4 (40.0)	0.211
CHEK2, *n* (%)	0 (0)	0 (0)	–	0 (0)	1 (10.0)	0.262
PALB2, *n* (%)	0 (0)	1 (3.7)	0.362	0 (0)	0 (0)	–
Bilateral tumor, *n* (%)	2 (9.1)	7 (25.9)	0.130	–	–	–
Simultaneous bilateral mastectomy, *n* (%)	20 (90.9)	24 (88.9)	0.816	12 (100.0)	10 (100.0)	–
Type of mastectomy						
NSM, *n* (%)	12 (54.6)	6 (22.2)	**0.020**	11 (91.7)	9 (90.0)	0.892
SSM,* n* (%)	10 (45.5)	21 (77.8)	**0.020**	1 (8.3)	1 (10.0)	0.892
Breast cancer						
Current BC, *n* (%)	13 (59.1)	12 (44.4)	0.308	–	–	–
Past BC, *n* (%)	9 (40.9)	15 (55.6)	0.308	–	–	–
Breast cancer stage						
DCIS	2 (9.1)	2 (7.4)	0.831	–	–	–
Early-stage BC, *n* (%)	15 (68.2)	15 (55.6)	0.367	–	–	–
Locally advanced BC,* n* (%)	4 (18.2)	10 (37.0)	0.146	–	–	–
Metastatic BC,* n* (%)	1 (4.6)	0 (0)	0.263	–	–	–
Chemotherapy						
Neoadjuvant,* n* (%)	10 (45.5)	18 (66.7)	0.136	–	–	–
Adjuvant,* n* (%)	11 (50.0)	17 (63.0)	0.362	–	–	–
Radiotherapy						
Neoadjuvant,* n* (%)	0 (0)	2 (7.4)	0.192	–	–	–
Adjuvant,* n* (%)	10 (45.5)	14 (51.9)	0.656	–	–	–
Positive family history of breast or ovarian cancer,* n* (%)	12 (54.6)	20 (74.1)	0.153	7 (58.3)	6 (60.0)	0.937
Smoker,* n* (%)	1 (4.6)	0 (0)	0.263	1 (8.3)	2 (20.0)	0.427

Bold, significant result (p < 0.05); DCIS, ductal carcinoma in-situ

### Effects of breast cancer status

The different stages of BC were balanced between groups, with early stage BC in the majority of cases. The amounts of patients with current or past BC, chemotherapy, or radiotherapy were also balanced between groups (Table 1**)**.

The BC status affected the type of mastectomy performed. Mutation carriers without BC mainly underwent NSM (90.9%, 20/22 vs. 36.7%, 18/49 in the BC group, *p* < 0.001), while skin-sparing mastectomy (SSM) was significantly more often performed in high-risk patients with BC (63.3%, 31/49 vs. 9.1%, 2/22, *p* < 0.001). All mutation carriers without BC underwent reconstruction (*n* = 44 breasts) after bilateral simultaneous prophylactic mastectomy (100%). In the BC group (*n* = 58 breasts with BC, *n* = 40 breast without BC), concurrent bilateral mastectomy was performed in 89.8% (44/49).

Logistic regression revealed that increasing age (OR = 1.076, 95% CI 1.017–1.139; *p* = 0.011), immediate reconstruction (OR = 4.395, 95% CI 1.349–14.324; *p* = 0.014), and medium-to-large breast size (OR = 3.883, 95% CI 1.107–13.613; *p* = 0.034) were predictive of ABR compared to IBR. In contrast, PMRT, NSM, and BC status were not significantly associated with ABR in the study (Table 2**)**.

**Table 2:** Binary logistic regression of variables associated with ABR in genetic susceptible patients

	*B*	SE	Wald	df	*p* value	Exp (*B*)	95% CI for Exp (*B*)	
							Lower	Upper
Age	0.073	0.029	6.448	1	**0.011**	1.076	1.017	1.139
Immediate reconstruction	1.480	0.603	6.032	1	**0.014**	4.395	1.349	14.324
Medium-to-large breast size	1.357	0.640	4.492	1	**0.034**	3.883	1.107	13.613
PMRT	0.576	0.711	0.657	1	0.418	1.779	0.442	7.167
NSM	− 0.470	0.700	0.451	1	0.502	0.625	0.158	2.465
Breast cancer	− 0.275	0.794	0.120	1	0.729	0.760	0.160	3.604
Intercept	− 4.436	1.813	5.988	1	0.014	0.012		

Bold, significant result (*p* < 0.05); SE, standard error; Exp (*B*), exponential value of B/odds ratio (OR); CI, confidence interval

### Impact of adjuvant radiotherapy

With regard to all BC cases with indication for PMRT, irradiation was equally performed after SSM (45.0%, 18/40) and NSM (38.9%, 7/18), *p* = 0.684. Although PMRT was not predictive of ABR, PMRT was significantly more often associated with SSM in the ABR-group (87.5%, 14/16) than with NSM (12.5%, 2/16), *p* = 0.021. In addition, irradiation of the TE in the IBR-group was significantly more often conducted (77.8%, 7/9) than after DTI (22.2%, 2/9), *p* = 0.005, while PMRT was significantly more common after immediate flaps in the ABR-group (75.0%, 12/16), *p* = 0.011.

### Impact of breast size

Among the factors analyzed, increasing age was identified as a significant predictor of ABR. In concordance, patients with ABR were significantly older than those with IBR, irrespective of breast size. Regarding BMI, patients with medium-to-large breasts exhibited significantly higher BMI values (26.8 ± 4.3 kg/m^2^) than those with smaller breasts (20.9 ± 2.2 kg/m^2^), *p* < 0.001. Similarly, the suprasternal notch-to-nipple distance (SSN:N) was markedly greater in medium-to-large breasts (26.8 ± 3.5 cm) compared to the small breast size group (20.3 ± 2.1 cm), *p* < 0.001. The mean mastectomy weight per breast was 256 ± 88 g for patients with small breasts and 667 ± 192 g for those with medium-to-large breasts, *p* < 0.001. Correspondingly, the implants selected for medium-to-large-breasted women were significantly larger than those for small-breasted patients, *p* = 0.004. The mean flap weight also increased proportionally with breast size, *p* < 0.001 (Table 3).

**Table 3:** Patients’ specifics and timing of breast reconstruction in genetic high-risk patients with small vs. medium-to-large breasts

	Small breast size group (*n* = 72 reconstructions)			Medium-to-large breast size group (*n* = 70 reconstructions)			
	IBR (*n* = 42)	ABR (*n* = 30)	*p* value	IBR (*n* = 26)	ABR (*n* = 44)	*p* value	*p* value
Age (years)	42.6 (28–65)^*^	50.6 (28–75)	**0.033**	39.2 (21–55)^*^	49.3 (35–70)	**0.005**	^*^0.320
BMI, *kg/m*^*2*^	20.6 (17–25)^*^	21.3 (18–25)^+^	0.413	25.5 (20–40)^*^	27.6 (20–35)^+^	0.180	^*^0.001, ^+^** < 0.001**
SSN:N, *cm*	20.5 (18–24)^*^	20.1 (16–25)^+^	0.710	26.5 (20–34)^*^	26.9 (22–35)^+^	0.810	^*,+^** < 0.001**
Mastectomy weight, *g*	256 ± 89^*^	255 ± 91^+^	0.972	606 ± 183^*^	703 ± 192^+^	0.152	^*,+^** < 0.001**
Implant volume, *cc*	333 ± 109	–	–	461 ± 97	–	–	**0.004**
Flap weight, *g*	–	354 ± 128	–	–	607 ± 181	–	** < 0.001**
Type of Mastectomy							
NSM,* n* (%)	36 (85.7)^*^	10 (33.3)	** < 0.001**	10 (38.5)^*^	20 (44.5)	0.568	^*^ < 0.001
SSM,* n* (%)	6 (14.3)^*^	20 (66.7)	** < 0.001**	16 (61.5)^*^	24 (55.6)	0.568	^*^ < 0.001
Timing of IBR							
Direct-to-implant (DTI),* n* (%)	17 (40.5)	–	–	5 (19.2)	–	–	0.069
Prior tissue expander,* n* (%)	25 (59.5)	–	–	21 (80.8)	–	–	0.069
Timing of ABR							
Immediate flap,* n* (%)	–	23 (76.7)	–	–	26 (59.1)	–	0.117
Delayed-immediate flap,* n* (%)	–	3 (10.0)	–	–	16 (36.4)	–	**0.011**
Delayed flap,* n* (%)	–	4 (13.3)	–	–	2 (4.6)	–	0.174
without prior recon., *n* (%)	–	2 (6.7)	–	–	0 (0)	–	0.083
with failed IBR,* n* (%)	–	2 (6.7)	–	–	2 (4.6)	–	0.692
Prior breast surgery							
Aesthetic augmentation, *n* (%)	4 (9.5)^*^	0 (0)	0.082	0 (0)^*^	2 (4.6)	0.270	^*^0.105
Reduction mammoplasty,* n* (%)	0 (0)^*^	0 (0)	–	7 (26.9)^*^	2 (4.6)	**0.007**	^*^ < 0.001
Breast conserving surgery,* n* (%)	5 (11.9)^*^	2 (6.7)	0.460	0 (0)^*^	4 (9.1)	0.113	^*^0.068

Bold, significant result (*p* < 0.05); *, *p* value of selected subgroup comparison of IBR-groups; +, *p* value of selected subgroup comparison of ABR-groups

A subgroup analysis revealed that preoperative breast size influenced the type of mastectomy performed within the IBR-group. Patients with smaller breasts who underwent IBR were significantly more likely to have NSM (85.7% vs. 38.5%, *p* < 0.001), whereas SSM was preferred in larger breasts within the IBR-group (61.5% vs. 14.3%, *p* < 0.001). Among patients with smaller breasts, NSM was significantly preferred in IBR over ABR (85.7% vs. 33.3%, *p* < 0.001), while SSM was more frequent in the ABR-group (66.7% vs. 14.3%, *p* < 0.001). In the context of ABR, the proportion of NSM and SSM procedures was not associated with preoperative breast size, *p* = 0.297.

### Timing of reconstruction in relation to breast size

**Fig. 1:**
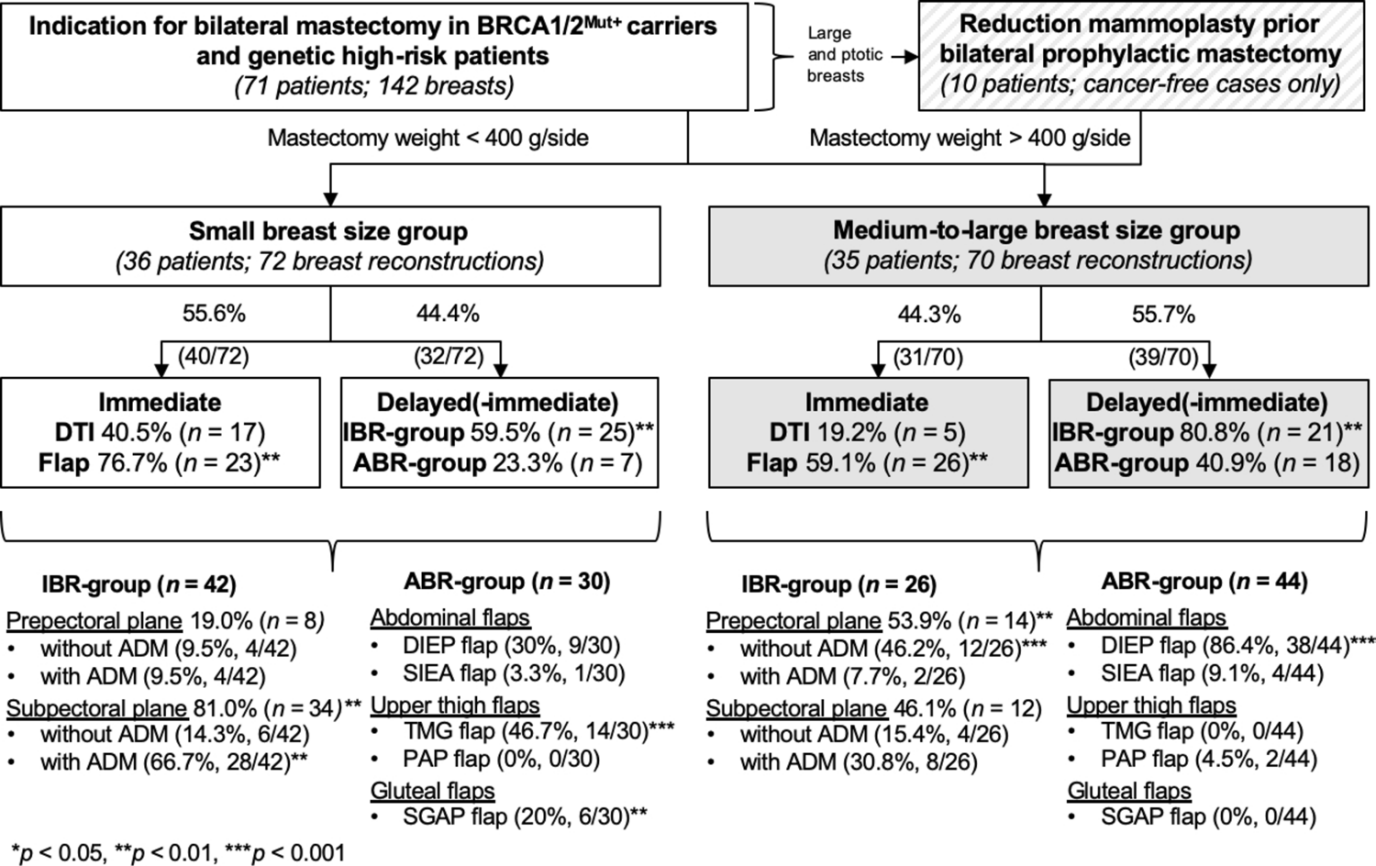
Study flowchart demonstrating the breast size-dependent reconstructive algorithm

DTI was performed in 40.5% (17/42) of cases with small breast size, whereas only 19.2% (5/26) with larger breasts underwent DTI, *p* = 0.069. TE placement was significantly more frequent in IBR than ABR for both small breasts (59.5%, 25/42 vs. 10.0%, 3/30, *p* < 0.001) and medium-to-large breasts (80.8%, 21/26 vs. 36.4%, 16/44, *p* = 0.001). Overall, larger preoperative breast size was associated with increased TE placements in both IBR (*p* = 0.069) and ABR (*p* = 0.011), Table 3. Across the entire cohort, immediate flaps were quantitatively superior to DTI (*p* < 0.01), while TE/implant-based reconstructions were more frequent in the "delayed(-immediate)" group (*p* < 0.01), Fig. 1.

### Breast size-dependent reconstruction

Comparative analysis of reconstruction techniques revealed distinct differences based on breast size. The breast size-dependent reconstructive algorithm is depicted in Fig. 1. Within the IBR-group, patients with small breast sizes predominantly received subpectoral implants (81.0%, *p* = 0.003) with ADM (66.7%, *p* = 0.004). Conversely, prepectoral implants (53.9%, *p* = 0.003) without ADM (46.2%, *p* < 0.001) were preferred for medium-to-large breasts.

**Fig. 2:**
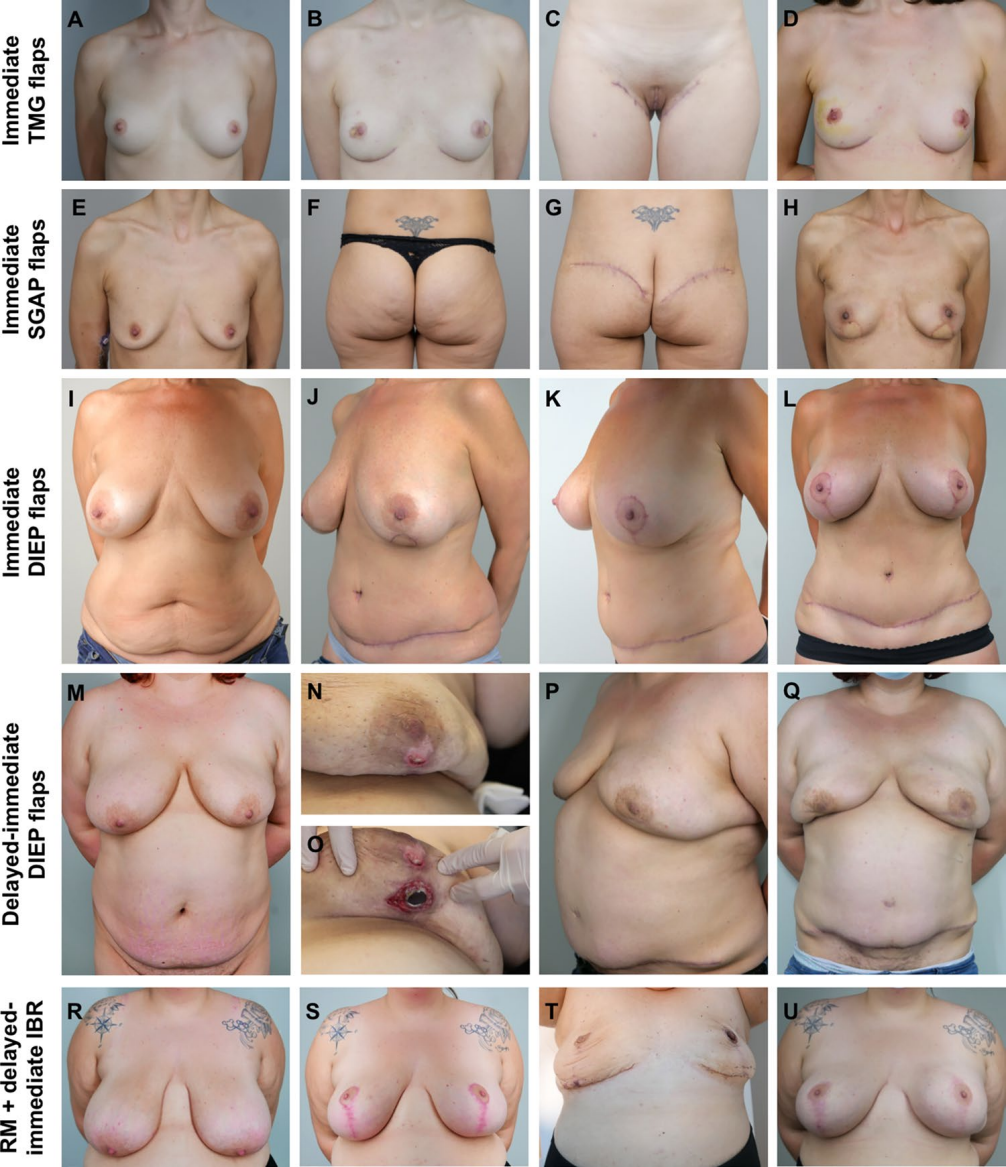
Preoperative conditions and postoperative results at 12-month follow-up after NSM and **A–D** bilateral immediate TMG flaps with **D** postreconstructive removal of the monitor islands, **E–H** immediate SGAP flaps, **I–L** immediate DIEP flaps with **K–L** postreconstructive resection of the monitor islands and bilateral mastopexy, **M–Q** delayed–immediate DIEP flaps with **N–O** tissue expander-associated complication, and **R–S** reduction mammoplasty prior **T** BPM and two-stage tissue expander/ **U** implant reconstruction

All transverse myocutaneous gracilis (TMG) flaps (46.7%, *p* < 0.001; Fig. 2A–D) and all superior gluteal artery perforator (SGAP) flaps (20.0%, *p* = 0.002; Fig. 2E–H) were exclusively performed in patients with small breasts and mastectomy weights below 400 g per side. In contrast, the DIEP flap was preferred in the vast majority of cases involving larger breasts (86.4%, *p* < 0.001; Fig. 2I-Q).

Analysis of breast surgeries performed prior to mastectomy and breast reconstruction revealed no statistically significant differences in the rates of aesthetic augmentation or breast conserving surgery. However, RM prior to mastectomy was more prevalent among patients with larger breasts (Table 3) and significantly more frequent prior IBR (Fig. 2R–U) than preceding ABR (26.9 vs. 4.6%), *p* = 0.007.

### Complications after tissue expander

All TE placements in the study (*n* = 65) were performed as delayed–immediate reconstructions. All expander-related complications and revisions are displayed in Table 4. Infection with subsequent TE loss occurred in 13.5% of cases in the medium-to-large breast size group (Fig. 2M–Q), while no TE loss was observed in the small breast size group, *p* = 0.043. All other complications remained statistical insignificant in the subgroup comparison. The sum of expander-related revisions requiring additional surgery was highly significantly increased (*p* = 0.003) in the medium-to-large breast size group.

**Table 4:** Complications after immediate tissue expander reconstruction of delayed–immediate cases in small vs. medium-to-large breasts

	Small breasts (*n* = 28)	Medium-to-large breasts (*n* = 37)	*p* value	All expanders (*n* = 65)
Expander-related complications				
Hematoma,* n* (%)	0 (0)	0 (0)	–	0 (0)
Seroma,* n* (%)	0 (0)	0 (0)	–	0 (0)
Capsular contracture, *n* (%)	3 (10.7)	4 (10.8)	0.990	7 (10.8)
Dislocation of expander,* n* (%)	2 (7.1)	1 (2.7)	0.398	3 (4.6)
Thinning of breast envelope,* n* (%)	2 (7.1)	2 (5.4)	0.773	4 (6.2)
Partial necrosis after SSM,* n* (%)	0 (0)	0 (0)	–	0 (0)
Partial necrosis after NSM,* n* (%)	0 (0)	2 (5.4)	0.211	2 (3.1)
Infection with expander loss, *n* (%)	0 (0)	5 (13.5)	**0.043**	5 (7.7)
Total, *n* (%)	7 (25.0)	14 (37.8)	0.273	21 (32.3)
Expander-related complications requiring additional surgery				
Pocket change,* n* (%)	0 (0)	1 (2.7)	0.381	1 (1.5)
Expander repositioning, *n* (%)	0 (0)	1 (2.7)	0.381	1 (1.5)
Expander exchange,* n* (%)	0 (0)	3 (8.1)	0.123	3 (4.6)
Expander removal,* n* (%)	0 (0)	3 (8.1)	0.123	3 (4.6)
New expander implantation,* n* (%)	0 (0)	2 (5.4)	0.211	2 (3.1)
Total, *n* (%)	0 (0)	10 (27.0)	**0.003**	10 (15.4)
Expander-related revisions during expander-to-implant exchange				
Pocket change,* n* (%)	2 (7.1)	0 (0)	0.099	2 (3.1)
Capsulotomy,* n* (%)	3 (10.7)	1 (2.7)	0.183	4 (6.2)
Capsulectomy,* n* (%)	0 (0)	3 (8.1)	0.123	3 (4.6)
Lipofilling, *n* (%)	2 (7.1)	2 (5.4)	0.773	4 (6.2)
Total, *n* (%)	7 (25.0)	5 (16.2)	0.381	13 (20.0)

Bold, significant result (p < 0.05)

### Impact of prior reduction mammoplasty

Twenty-eight immediate TE reconstructions were performed in patients with medium-to-large breasts. In contrast, nine elective breast reductions were performed prior to mastectomy with mean reduction weight of 392 ± 113 g. Prior RM was only performed in cancer-free breasts (Fig. 2R–U). In these cases, the subsequent mastectomy weight was 757 ± 219 g. In total, these patients received a removal of 1149 ± 300 g breast tissue, and the breast reduction comprised more than a third (34.5 ± 5.5%) of the total weight that has been removed. RM prior to TE reconstruction significantly reduced the amounts of complications requiring additional surgery from 35.7% (10/28) to 0% (0/9), *p* = 0.036.

### Complications after IBR

All events after IBR are presented in Table 5. Specific differences in elective revisions were only seen in the rate of pocket changes (*p* = 0.009) and lipofillings (*p* = 0.026), which were both significantly elevated in larger breasts. Finally, the total amount of elective revisions and NAC reconstructions were highly significantly increased in the medium-to-large breast size group, *p* < 0.001. The latter coincides with the amounts of SSM performed in the IBR-group (compare Table 3).

**Table 5:** Complications and elective revisions after IBR in small vs. medium-to-large breasts

	Small breasts (*n* = 42)	Medium-to-large breasts (*n* = 26)	*p* value	All IBRs (*n* = 68)
Complications after IBR				
Hematoma,* n* (%)	0 (0)	0 (0)	–	0 (0)
Seroma,* n* (%)	0 (0)	0 (0)	–	0 (0)
Capsular contracture, *n* (%)	4 (9.5)	4 (15.4)	0.466	8 (11.8)
Implant dislocation,* n* (%)	2 (4.8)	0 (0.0)	0.259	2 (2.9)
Rippling, *n* (%)	2 (4.8)	2 (7.7)	0.618	4 (5.9)
Scarring, *n* (%)	1 (2.4)	0 (0)	0.428	1 (1.5)
Thinning of breast envelope,* n* (%)	2 (4.8)	2 (7.7)	0.618	4 (5.9)
Partial necrosis after SSM,* n* (%)	0 (0)	0 (0)	–	0 (0)
Partial necrosis after NSM,* n* (%)	0 (0)	0 (0)	–	0 (0)
Erythema after irradiation,* n* (%)	1 (2.4)	0 (0)	0.428	1 (1.5)
Implant loss due to infection, *n* (%)	3 (7.1)	0 (0)	0.163	3 (4.4)
Total, *n* (%)	15 (35.7)	8 (30.8)	0.675	23 (30.9)
Elective revisions after IBR				
Implant exchange with				
Capsulotomy,* n* (%)	0 (0)	0 (0)	–	0 (0)
Capsulectomy,* n* (%)	4 (9.5)	4 (15.4)	0.466	8 (11.8)
Pocket change,* n* (%)	0 (0)	4 (15.4)	**0.009**	4 (5.9)
Implantation of ADM,* n* (%)	2 (4.8)	0 (0)	0.259	2 (2.9)
Lipofilling, *n* (%)	4 (9.5)	8 (30.8)	**0.026**	12 (17.7)
Scar correction,* n* (%)	2 (4.8)	0 (0.0)	0.259	2 (2.9)
Dog-ear correction,* n* (%)	0 (0)	2 (7.7)	0.068	2 (2.9)
Conversion to free flap,* n* (%)	2 (4.8)	2 (7.7)	0.618	4 (5.9)
Total, *n* (%)	14 (33.3)	20 (76.9)	** < 0.001**	34 (50.0)
NAC reconstruction,* n* (%)	6 (14.3)	16 (61.5)	** < 0.001**	22 (32.4)

Bold, significant result (p < 0.05)

### Complications after ABR

All events after ABR are listed in Table 6. Complications after ABR occurred statistically independently of the breast size. Each of the listed elective revisions viewed individually did not achieve statistical significance; however, patients with mastectomy weights above 400 g were more likely to undergo elective revisions, *p* = 0.013.

**Table 6:** Complications and elective revisions after ABR in small vs. medium-to-large breasts

	Small breasts (*n* = 30)	Medium-to-large breasts (*n* = 44)	*p* value	All ABRs (*n* = 74)
Complications after free flap				
Flap recipient site				
Venous anastomosis, *n* (%)	1 (3.3)	2 (4.6)	0.795	3 (4.1)
Arterial anastomosis,* n* (%)	1 (3.3)	1 (2.3)	0.782	2 (2.7)
Hematoma,* n* (%)	3 (10.0)	2 (4.6)	0.359	5 (6.8)
Partial flap necrosis,* n* (%)	0 (0)	1 (2.3)	0.406	1 (1.4)
Fat necrosis, *n* (%)	0 (0)	2 (4.6)	0.237	2 (2.7)
Flap loss, *n* (%)	1 (3.3)	0 (0)	0.223	1 (1.4)
Subtotal, *n* (%)	6 (20.0)	8 (18.2)	0.845	14 (18.9)
Flap donor-site				
Wound dehiscence,* n* (%)	2 (6.7)	2 (4.6)	0.692	4 (5.4)
Seroma,* n* (%)	2 (6.7)	1 (2.3)	0.347	3 (4.1)
Hematoma,* n* (%)	1 (3.3)	0 (0)	0.223	1 (1.4)
Subtotal, *n* (%)	5 (16.7)	3 (6.8)	0.180	8 (10.8)
Partial necrosis of mastectomy flap				
NSM group, *n* (%)	1 (3.3)	0 (0)	0.223	1 (1.4)
SSM group,* n* (%)	0 (0)	1 (2.3)	0.406	1 (1.4)
Subtotal, *n* (%)	1 (3.3)	1 (2.3)	0.782	2 (2.7)
Total, *n* (%)	12 (40.0)	12 (27.3)	0.251	24 (29.7)
Elective revisions after free flap				
Lipofilling, *n* (%)	4 (13.3)	10 (22.7)	0.311	14 (18.9)
Scar correction,* n* (%)	3 (10.0)	6 (13.6)	0.638	9 (12.2)
Dog-ear correction abdomen,* n* (%)	0 (0)	5 (11.4)	0.056	5 (6.8)
Flap volume reduction,* n* (%)	0 (0)	2 (4.6)	0.237	2 (2.7)
Resection of monitor islands,* n* (%)	3 (10.0)	2 (4.6)	0.359	5 (6.8)
- with bilateral mastopexy,* n* (%)	0	2 (4.6)	0.237	2 (2.7)
Resection of fat necrosis,* n* (%)	0 (0)	2 (4.6)	0.237	2 (2.7)
Conversion to implant,* n* (%)	1 (3.3)	0 (0)	0.223	1 (1.4
Total, *n* (%)	11 (36.7)	29 (65.9)	**0.013**	40 (54.1)
NAC reconstruction,* n* (%)	20 (66.7)	24 (54.6)	0.297	44 (59.5)

Bold, significant result (p < 0.05)

## Discussion

The advantages of breast reconstruction are well described and include an increased quality of life and overall satisfaction [2, 17]. BPM in BRCA1/2^Mut+^ carriers contributes to a 90–95% risk reduction for BC [2, 18]. The diagnosis of BC in BRCA1/2^Mut+^ carriers has shown to affect the likelihood of postmastectomy reconstruction [3], and a relationship between patient demographics and type of reconstruction has been seen [2]. According to a previous retrospective study including 1945 patients after BPM 71% underwent IBR, and only less than 10% decided for autologous tissue [2]. Presently, the majority of bilateral reconstructions of genetically high-risk patients involve implants rather than autologous tissue and available data on BRCA1/2^Mut+^ carriers undergoing flap reconstruction remain scarce [3, 19]. In great contrast, the ratio of IBR to ABR at the investigated institution is 1:1 and equivalent for both prophylactic and oncological cases. Although arguably technically more challenging and operative time consuming [2], our presented data support bilateral autologous reconstructions as viable option to implant-based reconstruction especially in immediate cases, medium-to-large breasts, and after failed IBR.

### Impact of breast cancer status

During the study period of 2.5-years, nearly every sixth patient at the investigated institution was a BRCA1/2^Mut+^ carrier. More than two-third of these patients revealed history of BC. In particular, the BC status was reported to affect the rate of breast reconstructions after RRM in the sense that patients with past history of BC were less likely to undergo postmastectomy reconstruction, [3, 4] while increased reconstruction rates were observed in BRCA patients with current status of BC [3]. This study does not evaluate the rate of patients that do not undergo postmastectomy reconstruction, as all included patients were reconstructed. In concordance to previous reports, mutations carriers without BC preferred nipple-sparing to skin-sparing mastectomies [3, 20], while the latter were significantly more often performed in oncological cases. NSM offers high aesthetic outcomes by preserving the skin envelope and leaving the NAC intact, and is associated with improved satisfaction as well as increased psychosocial and sexual well-being [21–23]. Recent data support NSM in BRCA1/2^Mut+^ carriers and conclude its oncological safety to be at least equal to other types of mastectomy for preventing BC occurrence in genetic susceptible patients [21]. Nevertheless, higher complication rates due to the risk of nipple necrosis need to be considered [24], and PMRT has shown to be one of the most significant risk factors for complication in NSM [24, 25]. In the presented study, patients with BC significantly preferred NSM in the IBR-group, while SSM was quantitatively superior in ABR. Several factors may have contributed to this result including oncological reasons and patient preferences. Previous data revealed that worries about tumor recurrence and the wish for breast symmetry influenced the decision of patients on prophylactic mastectomy and reconstruction [16]. Patients with BC in the ABR-group were significantly older than those with IBR, leaving the suggestion that safety concerns of NSM in older patients and a relatively higher acceptance for NSM in younger patients may have led to the observed results. This discussion is in line with the other authors that have argued that the increased popularity of NSM has contributed to the acceptance for prophylactic mastectomy and reconstruction especially in young patients [20].

### Impact of inherited breast cancer genes

This study included mainly BRCA1/2^Mut+^ carriers as well as one PALB2 and one CHEK2 carrier. An increased risk for contralateral BC in carriers of germline pathogenic variants in those BC genes has been recently described [26]. Neither tumor recurrence nor new diagnosis of BC was observed during follow-up. Cancer risk-reducing surgeries were described for women with inherited breast cancer genes other than BRCA1/2 [27]. As more data on the reconstruction process can help to inform affected patients, the presented study included PALB2 and CHEK2 carriers intentionally. However, there is a lack of guidelines for a risk-appropriate care in these rare cases. To prevent overtreatment, additional risk factors for BC should be considered prior to cancer risk-reducing surgery [27].

### Immediate or staged breast reconstruction

DTI reconstructions had shown to be associated with lower complications than delayed–immediate approaches with or without PMRT, and with complication rates equal to those of autologous reconstructions [28]. However, PMRT after DTI, two-stage IBR, or immediate flap reconstruction remain controversially discussed [28]. In general, delayed reconstructions are preferred if PMRT is indicated [2], while there are data indicating superiority of irradiation after DTI [28]. Conversely, recent data reveal that irradiation of immediate flaps was associated with less risk of reconstructive failure and higher satisfaction compared to PMRT in cases of one- or two-stage IBR [29]. In concordance, genetic high-risk patients of the presented study requiring irradiation were more likely to undergo immediate flap reconstruction, while PMRT was preferred after immediate TE placement in IBR. Unfortunately, the sample size of the study was insufficient for evaluation of the impact of PMRT on the outcome. More studies are needed to clarify this finding in genetic susceptible patients.

### Impact of breast size on reconstruction

According to the logistic regression results, we found that age, immediate reconstruction, and breast size were associated with the indication for ABR but not breast cancer status, NSM, or indication for PMRT. Prior to this study, the effects of breast size on postmastectomy reconstruction in high-risk patients have been insufficiently investigated. In general, women with greater mastectomy weights have been shown to be more prone to complications and wound infection [8], and postmastectomy reconstruction of large-breasted patients has been recognized as a reconstructive challenge [8, 30]. Women with large breast size are associated with elevated BMI and considered to be suboptimal candidates for prosthetic as well as autologous reconstruction due to poor aesthetic outcomes and increased complications [9, 14]. Previously, pedicled flaps had been discussed for patients with large breast size and mastectomy weight specimens greater than 1000 g [8]. Advances in microsurgery nowadays offer free flap reconstructions to patients even with larger breasts and elevated BMI [15]. Available flaps have evolved over the last decade and the presented study aims to provide new data on reconstructive options for high-risk patients.

Previous publications have stratified patients into three groups according to the mastectomy weight [8, 9]. As the sample size of the present study was limited, it was necessary to reduce the breast size groups to two. In this way, however, a great selectivity for the donor-site availability was achieved. Nearly all patients with mastectomy specimens greater than 400 g underwent flap reconstruction with abdominal donor-site. On the contrary, all TMG and SGAP flaps were performed in patients with mastectomy weights below 400 g. Beyond that, two profunda artery perforator (PAP) flaps in case of larger breasts were necessary, as the patient has lost the abdominal donor-site by preceding cosmetic abdominoplasty. To our knowledge, this is the first study providing comparative data that demonstrates a breast size-dependent selection of the thigh and gluteal area as additional donor-sites for microvascular breast reconstruction in BRCA1/2, CHEK2, and PALB2 mutation carriers.

The type of mastectomy is also influenced by the breast size. With regard to small-breasted patients, NSM was more often performed in IBR, while quantities of SSM were superior in ABR. The amount of NSM in IBR decreased significantly with increasing breast size, while SSM are favored in these circumstances. NSM in large breasts bear increased risks for skin flap and NAC necrosis [30], and is therefore discussed to be favored in smaller breasts. However, NSM can be performed in women with cup size greater than C and more, ptosis grade 2 and more, and in absence of risk factors for nipple necrosis (e.g., age, skin incision, skin flap thickness, type of reconstruction, and smoking) with the use of skin reduction with/without free nipple grafting or delayed after reduction mammoplasty [31, 32].

In terms of IBR, patients of the study with smaller breasts more often underwent subpectoral reconstruction with ADM, while prepectoral reconstructions without ADM were preferred in larger breasts. Previous outcome analysis reported comparable complication rates for subpectoral reconstructions with ADM and the latter [33]. Prepectoral reconstructions have gained worldwide popularity in recent years, and several advantages have been reported especially in prepectoral TE/implant reconstructions without ADM after NSM, including benefits with regard to the breast shape, less expander-related morbidity, ease of reconstruction, and cost efficiency [34]. However, clinical outcomes after prepectoral reconstructions remain debated [35].

Independent of the breast size and BC status, the amount of delayed–immediate procedures was superior in IBR and additionally two-stage TE reconstructions were 3.5 times more often performed in flap reconstructions of larger breasts. Different aspects may have contributed to these findings, including patient and surgeon preferences, and described advantages, e.g., reducing the pressure on the mastectomy flap, easier selection of implants for expander-to-implant exchange, and the option for elective revisions during the latter [36]. The use of TE in staged microsurgical breast reconstruction has shown several benefits. As discussed above, flap radiation remains a current controversy [29, 37]. Moreover, delayed–immediate autologous reconstruction has the advantage of avoiding radiation of the flap, shorter surgery time, and the possibility for patients to change their choice to implants, which is known to occur commonly [37].

### Prior reduction mammoplasty

The benefits of staged breast reduction before NSM have been previously reported [38]. The case presented in Fig. 2N–O displays a major complication that significantly occurred in patients with medium-to-large breasts without prior reduction mammoplasty: wound dehiscence of the mastectomy flap with infection of the breast pocket and consecutive expander loss. Although all cases of the study with expander loss could have been successfully revised, large breasts reveal a reconstructive challenge with potential poor cosmetic outcome if patients decide not to undergo prior RM (Fig. 2P–Q). As elevated mastectomy weights are more prone to postoperative complications, this study could demonstrate a significant reduction of morbidity related to expansion in high-risk patients with larger breasts by prior RM.

### Complications and revisions

The overall complications after TE, IBR, and ABR were not significantly elevated in larger breasts. Complications associated with either SSM or NSM were insignificant in the group comparisons. However, elective revisions were increased in all reconstructive groups with mastectomy weights greater than 400 g, and above all, the TE explant rate was elevated. In IBR, patients with larger breasts were more likely to undergo postreconstructive pocket change, lipofilling, and NAC reconstruction. With regard to ABR, the sum of elective revisions (mainly corrections of scars, breast form, and volume; Fig. 2K–L), but not microsurgical complications or flap donor-site complications, were significantly elevated with increased breast size. Finally, patients with failed IBR converted to autologous tissue, as previously described [39].

Last, but not least, the presented data demonstrate that a breast size-dependent reconstructive algorithm can contribute to successful reconstruction rates and suggest that bilateral free flap breast reconstruction can be safely offered to genetic high-risk patients as viable option to IBR independent of the preoperative breast size. Further, resource availability and financial constraints can affect the choice of reconstruction. Autologous reconstruction, while associated with higher initial costs and surgical complexity, may be more cost-effective over time due to higher health care use rates for implant-based techniques in the long term [40].

### Limitations of the study

The study has several limitations that should be considered. While it provides comparative data on different reconstruction techniques stratified by breast size, it does not attempt to directly compare the surgical outcomes and complications of each reconstructive method. Each technique has distinct risk profiles and associated complications, which limits their comparability. Additionally, the study focuses on a relatively small group of genetic high-risk patients, restricting the generalizability of the findings. Another limitation is that it only includes patients who underwent breast reconstruction and does not analyze those who opted against reconstruction after bilateral mastectomy, which could provide further insights into patient decision-making and outcomes. Furthermore, the impact of breast size on various surgical and aesthetic outcomes requires further investigation in larger cohorts. Nevertheless, pooled data on breast reconstruction after BPM exist, but are restricted to selected general target variables, revealing an extended length of hospital stay, higher amounts of events requiring a return to the operating room, more readmissions within the first postoperative month, increased superficial incisional surgical site infections, wound-healing problems, and need for postoperative blood transfusion after ABRs compared to IBRs and patients with mastectomy only [2]. Given these factors, additional studies involving a broader population of genetic high-risk patients are necessary to further clarify the influence of breast size on surgical results and other relevant clinical parameters.

## Conclusions

The preoperative breast size of gene mutation carriers influences the postmastectomy reconstructive choice. TMG and SGAP flaps are suitable for reconstruction of high-risk patients with small breasts, while DIEP flaps are favored in larger breast sizes. No elevated incidence of overall complications with increasing breast size but an increased risk for elective revisions after either IBR or ABR in genetic susceptible patients need to be considered. Women with medium-to-large breasts reveal increased morbidity related to expansion and genetic high-risk patients may benefit from prior reduction mammoplasty.
